# Influence of Work Environment Characteristics on the Level of Professional Burnout of Healthcare Professionals in Northeastern Bulgaria

**DOI:** 10.3390/healthcare13202607

**Published:** 2025-10-16

**Authors:** Teodora Dimitrova, Tsvetelina Tarpomanova, Antoaneta Tsvetkova, Yana Tosheva, Velislava Venkova, Anna Todorova

**Affiliations:** 1 Department of Hygiene and Epidemiology, Faculty of Public Health, Medical University of Varna, 9002 Varna, Bulgaria; tdimitrovatvd@gmail.com; 2 Medical College, Medical University of Varna, 9002 Varna, Bulgaria; tsvetelina.tarpomanova@mu-varna.bg (T.T.); antoaneta.tsvetkova@mu-varna.bg (A.T.); 3 Faculty of Pharmacy, Medical University of Varna, 9002 Varna, Bulgaria; velislava.venkova@abv.bg; 4 Department of Organization and Economics of Pharmacy, Faculty of Pharmacy, Medical University of Varna, 9002 Varna, Bulgaria; anna.todorova@mu-varna.bg

**Keywords:** healthcare professional, master pharmacist, assistant pharmacist, public health inspector, burnout, work environment characteristics

## Abstract

**Background:** Burnout is a common occupational health risk among healthcare professionals. While the phenomenon has been studied in physicians and nurses, limited evidence exists regarding pharmacists and public health inspectors despite their critical role in ensuring safe medication use and protecting population health. The study aims to identify and analyze the influence of work environment characteristics on the level of professional burnout among master pharmacists, assistant pharmacists, and public health inspectors. **Methods:** A cross-sectional anonymous survey was conducted among 491 healthcare professionals (221 master pharmacists, 151 assistant pharmacists, and 119 public health inspectors). Burnout was assessed using the validated Maslach Burnout Inventory–Human Services Survey (MBI-HSS (MP)), covering three dimensions: emotional exhaustion (EE), depersonalization (DP), and personal accomplishment (PA). Work environment characteristics were examined across four domains: work tasks, stressors, occupational risks, and social environment. Data were analyzed using descriptive statistics and chi-square tests with IBM SPSS. **Results:** High levels of EE (66.6%) and DP (53%) were reported, while low PA was less frequent (6.7%). Significant factors associated with EE included time constraints (χ^2^ = 9.985; *p* < 0.01), workflow disruptions (χ^2^ = 23.987; *p* < 0.001), insufficient information (χ^2^ = 22.890; *p* < 0.001), and lack of recognition (χ^2^ = 16.498; *p* < 0.001). The social environment demonstrated the broadest impact, influencing all three burnout dimensions. **Conclusions:** The study found a risk of professional burnout among the surveyed groups which is associated with modifiable work environment characteristics. Preventive interventions aimed at promoting a supportive work environment could help mitigate this risk.

## 1. Introduction

Burnout, or “occupational burnout syndrome” is a condition that develops in response to the traumatic impact of stress from the work environment. Among the most vulnerable are employees in the healthcare sector, where the specificity of their work requires constant interaction and communication with patients and clients [1]. One of the main challenges facing healthcare systems, not only in Bulgaria but also in Europe and worldwide, is the severe shortage of human and financial resources. In conditions of health crisis, the excessive workload, time pressure and extraordinary stress among healthcare professionals are a prerequisite for the development of burnout syndrome [2]. The dynamic development of the healthcare system and the intensively changing work environment of healthcare professionals is a prerequisite for the emergence of a number of challenges in providing quality patient care and ensuring patient safety. This requires increased control over the healthcare services provided in the community. As a result, burnout affects both the providers of healthcare services and those monitoring their proper provision [3].

A poorly structured work environment intertwined with individual characteristics is a prerequisite for the occurrence of the phenomenon of professional burnout. According to the definition by the World Health Organization (WHO) in the International Classification of Diseases (ICD-11), professional burnout is a condition described as emotional, physical, and mental exhaustion observed in healthy people as a result of unmanaged work-related stress [4].

It is characterized by three dimensions: emotional exhaustion and depletion, cognitive detachment from work and a sense of negativity toward the work process, and reduced work efficiency.

Defined as a syndrome of emotional exhaustion, depersonalization, and reduced personal accomplishment, burnout not only undermines professional well-being among healthcare professionals but also threatens the quality and safety of healthcare delivery [5,6].

While the phenomenon has been widely studied among physicians and nurses, far less is known about its prevalence and determinants in other healthcare professionals, such as pharmacists and public health inspectors, whose roles are equally essential for health system effectiveness [7,8,9,10].

These professionals, though diverse in function, share a common set of challenges. Master pharmacists are responsible for the safe and effective use of medicines. Their duties include dispensing prescription medicines, ensuring compliance with legal and professional standards, managing drug therapy, providing counselling, monitoring drug therapy and potential drug interactions, and delivering pharmaceutical care [11,12].

Assistant pharmacists support master pharmacists in the delivery of pharmaceutical services. Their duties typically include counselling patients on the safe and effective use of over-the-counter (OTC) drugs and food supplements, providing self-care advice and guidance on minor health conditions, and preparing individualized formulations through extemporaneous compounding [13,14].

Public health inspectors work on the preventive and regulatory side of healthcare. They are responsible for safeguarding community health through inspections, disease prevention, risk assessments, and enforcement of health standards. Their work involves monitoring compliance in areas such as food safety, sanitation, environmental health, infectious disease control, and occupational health risks. They often interact with businesses, institutions, and the public to assess risks, provide guidance, and ensure adherence to regulations [15]. While not traditionally viewed as frontline healthcare providers, their responsibilities carry a direct impact on population health outcomes.

Despite the variation in duties, all three groups face high workloads, constant accuracy requirements, interpersonal demands, and responsibility for public health outcomes.

Work environment characteristics play a decisive role in shaping the risk of burnout. Factors such as time pressure, insufficient resources, disruptions in work processes, limited recognition, and lack of supportive communication can exacerbate stress and erode resilience [16,17]. In pharmacy practice, these stressors may increase the likelihood of dispensing errors or compromise patient counselling, while in public health inspection, they may lead to reduced vigilance or strained community relations [18,19].

Yet, research into how these specific work environment characteristics influence burnout among pharmacists and public health inspectors remains limited.

The study focuses on master pharmacists, assistant pharmacists, and public health inspectors because these healthcare professionals play critical roles in ensuring public health, medication safety, and effective healthcare delivery, yet they are underrepresented in burnout research.

There are limited findings regarding these three groups of healthcare professionals. Studies in the United States report burnout rates of over 50% among pharmacists with emotional exhaustion and depersonalization being the most common dimensions [20]. Burnout among community pharmacists has also been documented in the United Kingdom, where recent surveys report similarly high rates with emotional exhaustion as the predominant dimension [21]. Evidence further indicates that UK community pharmacists experience exceptionally high levels of burnout often exceeding those observed in hospital practice [22]. Recent research has also highlighted that pharmacy assistants and technicians, as vital members of pharmacy teams, are not exempt from the risk of burnout. Studies in Thailand have reported high levels of emotional exhaustion and depersonalization related to workload and limited recognition [23]. In addition, burnout among healthcare inspectors has also been documented. Findings from Greece and Australia indicate high levels of burnout in terms of emotional exhaustion and depersonalization as well as low levels of personal accomplishment [24,25].

To our knowledge, this is the first study in Bulgaria to simultaneously examine professional burnout among three distinct healthcare groups—master pharmacists, assistant pharmacists, and public health inspectors. The main contribution of the present study is to address this knowledge gap by analyzing the influence of modifiable work environment factors on burnout in these groups. By exploring roles that share overlapping responsibilities but differ in professional scope and organizational context, the study provides a unique perspective on how work environment characteristics contribute to burnout across complementary sectors of the healthcare system.

Empathy, altruism, and attachment in a professional environment, brought to extreme levels, determine emotional and cognitive impairments with more negative effects in the long term [26].

Workplace stress is one of the determinants of health [27]. It is necessary to create a favorable working environment in order to preserve the health of specialists, provide conditions for full performance of duties, and reduce the risk of errors [17,28,29]. This requires research into the work characteristics that directly influence occupational stress, which could serve as a basis for developing recommendations and training programs for the prevention of occupational burnout.

The study aims to identify and analyze the influence of work environment characteristics on the level of professional burnout among master pharmacists, assistant pharmacists, and public health inspectors.

## 2. Materials and Methods

### 2.1. Study Design

A cross-sectional descriptive study was performed using an anonymous self-administered questionnaire. Burnout levels were assessed with the validated Maslach Burnout Inventory–Human Services Survey (MBI-HSS (MP)), while work environment characteristics were evaluated across four domains: work tasks, stressors, occupational risks, and social environment. This design allows both the assessment of burnout prevalence and the analysis of its associations with workplace factors among pharmacists and public health inspectors.

The study included a total of 491 healthcare professionals (372 community pharmacists—221 pharmacists and 151 assistant pharmacists—and 119 public health inspectors) from the northeastern region of Bulgaria (the cities of Varna, Ruse, Dobrich, Shumen, Razgrad, Silistra, and Targovishte) in the period from November 2023 to December 2024.

The study was conducted using a direct, anonymous online questionnaire, following the principles of voluntary participation and confidentiality.

The created survey link was distributed by email with the support of designated representatives from relevant professional organizations: the Bulgarian Pharmaceutical Union (master pharmacists), the Bulgarian Association of Assistant Pharmacists (assistant pharmacists), and individuals appointed by the directors of the Regional Health Inspectorates (for RHI employees) by regions (the cities of Varna, Ruse, Dobrich, Shumen, Razgrad, Silistra, and Targovishte).

Prior to the distribution of the survey, written declarations of consent were obtained at the national level from the chairpersons of the Bulgarian Pharmaceutical Union and the Bulgarian Association of Assistant Pharmacists and at regional level from the chairpersons of the regional pharmaceutical chambers and the directors of the Regional Health Inspectorates by regions (the cities of Varna, Ruse, Dobrich, Shumen, Razgrad, Silistra, and Targovishte).

Prior to participation in the survey, all respondents were informed about the purpose of the study and the intended use of the collected data, after which they provided informed consent. The electronic format of the survey included the information provided for the participants and the informed consent form, both of which were mandatory to complete.

The respondents were required to confirm that they had read and understood the information and agreed to participate in the study. The participants were free to withdraw from the study at any time without any consequences. To control the completion of the filled questionnaires, revision was conducted prior to data entry to ensure the quality and reliability of the dataset.

No personal information was collected during the study, and participant responses were coded to ensure their anonymity.

### 2.2. Sample Selection

Official data from the registries of licensed pharmacists and assistant pharmacists practicing in northeastern Bulgaria were used for the selection of the sample of community pharmacists. The total number of pharmacists practicing in community pharmacies in northeastern Bulgaria was 1652 at the time of the study. The classical statistical formula for finite populations was applied in order to determine the minimum required sample size [30,31]. Thus, for a total population of *n* = 1652 pharmacists, assuming a 95% confidence interval and a ±5% margin of error, the minimum required sample size to ensure the representativeness of all pharmacists working in community pharmacies in northeastern Bulgaria was calculated to be *n* = 312 respondents. Within this population, the proportion of master pharmacists to assistant pharmacists is 63% (*n* = 1044) to 37% (*n* = 608). Therefore, 197 master pharmacists and 115 assistant pharmacists were required in order to achieve full representativeness within the sample of 312 community pharmacists.

A total of 372 community pharmacists were included in this study: 221 master pharmacists and 151 assistant pharmacists. Thus, the sample can be considered statistically justified and representative, allowing the conclusions and analyses to be valid for the entire studied population.

Sample Selection for Public Health Inspectors: According to the regulations of the Regional Health Inspectorates (RHIs), a total of 337 specialists work in the northeastern region, 130 of whom are public health inspectors [32]. This study included 119 public health inspectors, representing 91.54% of the total population and ensuring the reliability of the results.

The participants were selected on the basis of strictly defined inclusion and exclusion criteria.

Inclusion criteria:

- To be actively practicing in the northeastern region of Bulgaria (the cities of Varna, Ruse, Dobrich, Shumen, Razgrad, Silistra, and Targovishte).
- To be employed full time.
- To have more than six months of professional experience in their field of specialization.
- For pharmacists and assistant pharmacists: to be practicing in community pharmacies.
- For public health inspectors: to be employed at the Regional Health Inspectorates (RHIs).
- The participants were required to be members of the relevant professional organization: the Bulgarian Pharmaceutical Union (master pharmacists), the Bulgarian Association of Assistant Pharmacists (assistant pharmacists), public health inspectors to be RHI employees.
- Provided informed consent.

Exclusion criteria:

- Professionals practicing in other sectors outside of community pharmacies or the RHIs.
- Professionals practicing outside the territory of the northeastern region.
- Students, interns, pensioners.
- Specialists with prolonged absence from work (more than three months) due to illness, maternity leave, or other circumstances.
- Refusal or unwillingness to participate in the study.
- Detected incomplete questionnaires.
- Detected inconsistent answers.
- Detected unclear answers.

### 2.3. Description of the Research Tool

The study was conducted using a structured questionnaire provided by the researchers specifically for the purposes of this study. The questionnaire consisted of 3 parts:

1. Questions related to demographic characteristics: sex, age, professional experience, location of practice, education (5 questions);
2. A validated instrument for assessing burnout, the Maslach Burnout Inventory–Human Services Survey (MBI-HSS (MP), was used to evaluate professional burnout. The instrument assesses burnout through a combination of three components: emotional exhaustion (EE), depersonalization (DP), and reduced personal accomplishment (PA) [33].

The test consists of 22 statements, which were rated on a frequency scale from 0 to 6 (0—never; 1—once a year or less; 2—once a month; 3—a few times a month; 4—once a week; 5—a few times a week; 6—every day) for three components: emotional exhaustion, depersonalization and personal accomplishment. The summed scores for the three components are shown in Table 1.

High scores on emotional exhaustion and depersonalization and low scores on personal accomplishment were indicative of burnout.

3. Part III of the questionnaire was adapted directly from an internationally recognized guideline of the European Commission: “Risks to health and safety at work in the healthcare sector—Guide to prevention and good practice” [34]. The items included in Part III can be considered reliable and valid as they originate from a standardized and widely used instrument (45 questions).

Work-related characteristics were grouped as follows:

- Risk factors arising from work tasks such as too high qualitative and quantitative demands (e.g., patients, clients, or large affected groups in the community), time pressure and tight deadlines, information overload, conflicting work instructions given by immediate supervisors, etc.
- Stress factors arising from the role at the workplace such as insufficient competence, lack of professional experience, excessive responsibility, unclear assignment of tasks, lack of support and assistance, absence of recognition, etc.
- Environmental stress factors in the workplace, such as exposure to toxic substances, biological agents and needlestick injuries, complex technological systems that overload human cognitive capacity and decision making or exceed the ability to process and manage information, etc.;
- Risk factors arising from the social environment, such as poor psychosocial work environment, limited or ineffective communication, conflicts with managers and colleagues, frequent changes in the work environment, colleagues or field of work, structural changes, inadequate work–family balance, staff shortages, etc.;

To complete the entire questionnaire took respondents no more than 10–15 min. It contained 72 questions in total.

### 2.4. Statistical Methods

The obtained data are processed with the statistical package IBM SPSS, v.25 for windows by applying descriptive statistics, non-parametric tests (chi-square test for hypothesis testing). A *p*-value < 0.05 was considered statistically significant. The dependent variables are the three components of the Burnout Survey: emotional exhaustion (EE), depersonalization and dehumanization (DP), and impaired professional performance (PP), and the independent variables are occupational characteristics.

### 2.5. Ethical Approval

The study was conducted in accordance with the principles outlined in the Declaration of Helsinki and received ethical approval from the Research Ethics Committee of the Medical University of Varna with Report No. 135/28 September 2023.

## 3. Results

The survey includes 491 respondents, which were distributed as follows: 221 master pharmacists (45.01%), 151 assistant pharmacists (30.8%), and 119 public health inspectors (24.24%). The distribution of the surveyed medical specialists by gender, average age and place of practice—Varna (the largest regional city in northeastern Bulgaria) and outside Varna is presented in Table 2.

The difference of 2% is within the permissible deviation and reflects the centralization of administrative and inspection activities in the regional centers [32].

The distribution of the surveyed respondents across the three scales is presented in Table 3.

The results show that 327 (66.6%) of the respondents fall into the group with a high level of EE. A high level of DP is observed in 260 (53%) of the individuals studied. Low level of PA is observed in 33 (6.7%).

The results of the impact of work environment characteristics on the high levels of the three dimensions of the Maslach scale are presented in Table 4.

Lack of time statistically significantly affects the distribution of respondents into groups of demonstrated EE. In addition to EE, awareness of the need to make urgent decisions also statistically significantly affects the second dimension of the burnout scale—the degree of DP, but not the degree of PA (Table 4).

Disruptions and interruptions of professional activities are also a significant factor in the work process contributing to EE in the studied group. The presence of strict specific work performance requirements has a statistically significant effect only on the level of EE. Decision making without adequate information and sufficient supporting resources is identified as a risk characteristic.

A significantly higher proportion of the participants with high levels of EE reported the presence of conflicting demands. A lack of recognition for employees’ work was significantly more likely to be reported as a risk factor and less likely to be perceived as neutral among those with high levels of EE. A similar pattern was observed among respondents with high levels of DP (Table 4).

A high level of emotional strain was noted mainly by the respondents who identified unclear tasks, or tasks for which they lacked appropriate qualifications, as a risk characteristic. The same was pointed out also by those with high levels of DP.

In the group with high EE, a large percentage complained about exclusion from risk assessment and planning processes, whereas a significantly smaller proportion considered this aspect of workplace organization to be of minor importance. Among participants with high DP, a higher proportion also regarded this factor as important at the workplace.

Additionally, awareness of physical and ergonomic workplace hazards significantly influenced the distribution of medical professionals across EE groups. These factors were more frequently rated as important also in the groups with high DP and high levels of PA (Table 4).

Lack of support from colleagues and organizational leaders was reported by more than one third of the participants and disregarded by more than half of those with high levels of EE. This factor was also more frequently observed among participants with pronounced DP but not among those with PA (Table 4).

Psychosocial work environment, as a source of mental and emotional strain, was identified as a significant factor impacting EE. Among participants who considered the organization at work important, a higher proportion exhibited high levels of EE compared to those who considered it unimportant or moderately important. Conversely, among participants for whom the psychosocial work environment was not a particularly significant factor, the majority had moderate or low levels of EE. This factor also significantly influenced the distribution of participants across groups with high DP and PA (Table 4).

The need for social and communication skills within the teams was also significantly more frequently recognized by the participants with EE. A similar reliable association was observed in the groups with high DP and PA (Table 4).

## 4. Discussion

The demographic profile of the sample indicates a prevalence of female participants, particularly among assistant pharmacists and public health inspectors. These findings are consistent with the trends reported in the pharmaceutical and public health sectors in Europe, where women constitute the majority of the healthcare workforce [35]. Studies on burnout also suggest that female healthcare professionals are more vulnerable and more likely to experience EE partially because of the need to balance professional responsibilities with family obligations [36].

The age distribution indicated that assistant pharmacists comprised the youngest group. Other studies have reported higher levels of burnout among younger professionals because of the lower stress-coping mechanisms and fewer years of professional experience [37].

In the studied sample, the majority of the participants experienced EE (66.6%), and more than half of them (53%) exhibited high levels of DP. These findings are consistent with international studies reporting burnout levels exceeding 50% among healthcare professionals and public servants [38,39,40]. The results showing predominantly elevated levels of EE among professionals working in dynamic environments with high emotional demands are also confirmed [1,41,42,43].

Despite the low scores of PA observed in this study (6.7%), particular attention should be paid as it is often considered the final stage of burnout and may signal forthcoming personnel and organizational challenges. The identified pattern corresponds to the stages described by Maslach, according to which the effects on work capacity typically manifest at a later phase or may be temporarily compensated [44].

Some studies conducted among public health inspectors during the COVID-19 pandemic showed a sixfold increase in the percentage of individuals reporting high levels of EE (rising from 12.9% in 2019 to 75.4% in 2021), a substantial increase in DP (from 16.1% to 41%), as well as a marked rise in the cases of reduced PA (from 54.8% to 70.5%) [42]. The trend of elevated levels of EE and DP within this professional group has persisted to the present time.

Some of the task-related risk factors associated with EE and DP are insufficient time to complete a task, frequent interruptions in the workflow, inadequate or unclear information, and contradictory demands (between strict deadlines and the requirement to maintain high quality).

The stressors related to the work role and identified as predisposing factors for the development of professional burnout include the lack of recognition and the assignment of tasks for which the individuals have insufficient competence. Similar studies have demonstrated higher levels of burnout among younger professionals, particularly those who do not have access to mentors and who are frequently loaded with responsibilities that exceed their level of competence [45,46].

Other studies have also indicated that insufficient recognition from supervisors is associated with decreased job satisfaction among the pharmacists practicing in community pharmacies. Psychosocial risk factors such as excessive workload, pressure, stress, and direct interference from managers have been identified as key determinants of burnout, affecting as many as 87% of public health inspectors in Greece. These factors have a strong relationship with the level of EE (r = 0.513) [47].

Among the four categories of workplace stressors examined, the social environment stands out as a significant determinant of burnout across all three dimensions of the MBI (EE, DP, PA). The lack of support from managers and colleagues, the heightened psycho-emotional tension and the insufficient social and communication skills are strongly associated with higher levels of professional burnout. Other studies emphasize the protective role of supportive professional relationships in mitigating the adverse effects of occupational stress [48]. Similar results have been reported by physicians and nurses, where collegial support has proven to be a critical factor in overcoming EE [49].

The research regarding pharmacists and public health inspectors in this field remains scarce, which underscores the originality of this study.

The results indicate that even non-clinical groups of healthcare professionals are affected by different levels of risk for professional burnout. All types of health personnel engaged in safeguarding personal and public health should receive adequate promotion and mental health prevention at the workplace.

The primary factors contributing to work-related stress for master pharmacists include a high volume of administrative duties and responsibilities, the need for heightened attention when processing prescriptions, dispensing medications, providing consultations, and maintaining continuous contact with the patients, as well as a lack of sufficient communication skills. Organizational problems such as time constraints and workflow interruptions were also reported to have an impact. These results are consistent with previous international studies in pharmacy practice, which have repeatedly demonstrated that lack of time, strict deadlines and workflow interruptions can cause burnout and increase the risk of medication dispensing errors. A previous study among pharmacists in Bulgaria found that a lack of time for providing pharmaceutical care is associated with EE, DP, and reduced PA. Work overload not only leads to burnout but also to professional dissatisfaction, conflicts with patients and difficulties in the communication with other healthcare professionals [50]. Social and communication skills are a key competency for pharmacists and form the foundation for effective collaboration with patients in the pharmacy setting [51,52]. Interruptions in the workflow and frequent shifts of attention from one task to another require enhanced concentration and may cause administrative and clinical errors. This is confirmed by data in the literature that the high levels of professional burnout are associated with reduced cognitive performance and work-related errors [19].

The psychosocial risk factors are a key determinant also for public health inspectors (87%) in Greece, showing a strong correlation with the level of EE (r = 0.513, *p* < 0.01) and remaining a significant predictor of burnout even after adjustment for demographic factors (R2 = 0.449) (β = 0.455, *p* < 0.001) [40,41].

The psychosocial hazards in the workplace have been recently recognized as a major contemporary challenge to occupational health and safety. Work-related stress is defined as the harmful physical and emotional response caused by a mismatch between the perceived demands and the individual’s resources and capabilities to cope with those demands, often leading to a higher incidence of employee absenteeism due to illness [53].

High workload is also recognized as a factor conducive to burnout. This finding was reported in a survey of 116 public health inspectors in Bulgaria conducted between 2020 and 2021. The respondents clearly reported increased workload in their daily activities, pointing to the continuous increase in work assignments as the primary cause (χ^2^ = 19.312, *p* = 0.004) [42].

The excessive workload, time pressure and insufficient support further exacerbate the work environment. In the United States, half of public servants (49%) reported that they have considered leaving their positions due to burnout resulting from these factors, and some even ceased performing their work duties. Time pressure and excessive workload often exceed employees’ capacity to cope [40].

These statements reveal that there is a problem not only for individuals but also an organizational risk. This is further supported by other work characteristics and their association with burnout levels.

As early as 2008, nearly half of public sector employees in Bulgaria (48.1%) point out that these are a major stress factor [54].

Work-related stress among healthcare professionals, arising from the execution of their specific roles, is particularly intensified during health crises, such as the COVID-19 pandemic [45]. The experience from the recent pandemic indicates that burnout significantly affects both those responsible for implementing new regulations and exercising control as well as the healthcare professionals who have direct contact with patients [55,56].

### Limitations of the Study

- Limitations arise from the study design, which was cross-sectional:
  1. The design offers a snapshot of the current state but does not permit longitudinal analysis of how workplace environment factors lead to changes in burnout levels in the long term.
  2. The study is limited to northeastern Bulgaria, which may limit the possibilities to generalize the findings.
  3. The findings obtained for the region may not be fully applicable to countries with different organizational structures of their pharmaceutical and healthcare systems.
- The studies conducted in Bulgaria among the examined healthcare professionals are too limited, which does not allow for comparisons with previous research.
- The applied statistical analysis allows for either rejecting or confirming the hypothesis of a relationship between two categorical variables without establishing the strength or direction of the observed association. Future studies could include correlation or regression analyses in order to establish the strength of these associations.
- The response rate of participants could not be calculated, as the survey was anonymous and voluntary and the exact number of recipients was unknown.
- The sample size did not allow for subgroup analyses by professional role or by region (Varna vs. outside Varna). Future studies with larger and more evenly distributed samples should examine burnout variations by profession and region. In addition, future research should focus on expanding the study to other regions, developing intervention strategies to reduce burnout in these populations and monitoring their impact over time.

## 5. Conclusions

The study found a risk of professional burnout among the surveyed groups of master pharmacists, assistant pharmacists and public health inspectors in northeastern Bulgaria because of the fact that more than half of the respondents exhibit high levels of EE and DP (based on the Maslach Burnout Inventory). The following organizational factors of the work environment had the strongest impact on the burnout dimensions: time constraints, workflow interruptions, insufficient information and lack of recognition. The social environment (insufficient colleague support, poor communication skills and high psycho-emotional stress) emerged as a key predictor across all three burnout dimensions.

This study indicates that professional burnout among pharmacists and public health inspectors is primarily associated with modifiable characteristics of the work environment. Interventions targeting organizational efficiency, the improvement of communication skills, the recognition of professional efforts, the development of strategies for mental health prevention, the introduction of specialized trainings and the cultivation of a supportive social environment may mitigate the risk of burnout and promote the sustainability and quality of the delivered healthcare services.

## Figures and Tables

**Table 1:** The Maslach Burnout Inventory scores.

Levels	Scores		
	Low	Medium	High
Emotional exhaustion (EE)	0–15	16–24	≥25
Depersonalization (DP)	0–5	6–10	≥11
Reduced personal accomplishment (PA)	Above 37	36–31	Below 31

**Table 2:** Demographic characteristics of the subjects studied.

Characteristic		Master of Pharmacy		Assistant Pharmacist		Public Health Inspector	
		n	%	n	%	n	%
Gender	Female	171	77%	132	87%	102	86%
	Male	50	23%	19	13%	17	14%
Age (average)		35.9+/−0.8		32.05+/−0.8		40.87+/−1.3	
City	Varna	147	67%	112	75%	58	49%
	Outside Varna	74	33%	39	35%	61	51%
Total		221	45%	151	41%	119	24%

**Table 3:** Distribution of the studied individuals in the groups of high degree of EE, DP and PA.

Scale	*n*	%
High level of EE	327	66.6%
High level of DP	260	53%
High level of PA	33	6.7%

**Table 4:** Distribution of risk characteristics from the work process and the work environment in the groups of high degree of EE, DP and PA.

Risk Characteristic of the Work Process or Work Environment		High Level of EE		High Level of DP			High Level of PA		
		Number (%)	χ^2^, *p*	Number	%	χ^2^, *p*	Number	%	χ^2^, *p*
**Risk factors resulting from work tasks**									
The lack of time	Yes	*n* = 259 (79.2%)	9.985 *p* < 0.01	*n* = 211	(56.6%)	8.2 *p* < 0.05	*n* = 247 (66.2%)		2.904 *p* > 0.05
	No	*n* = 68 (20.8%)		*n* = 49	(41.5%)		*n* = 84 (71.2%)		
Disruptions and interruptions of professional activities	Yes	*n* = 208 (74%)	23.987 *p* < 0.001	*n* = 171 (60.9%)		19.432 *p* < 0.001	*n* = 178 (63.3%)		10.118 *p* < 0.01
	No	*n* = 119 (56.7%)		*n* = 89 (42.4%)			*n* = 153 (72.9%)		
The presence of strict specific requirements	Yes	*n* = 286 (67.3%)	8.087 *p* < 0.05			*p* > 0.05			*p* > 0.05
	No	*n* = 41 (62.1%)							
Without proper information	Yes	*n* = 200 (76%)	22.89 *p* < 0.001	*n* = 158 (60.1%)		13.297 *p* < 0.001			*p* > 0.05
	No	*n* = 127 (55.7%)		*n* = 102 (44.7%)					
Conflicting requirements (e.g., conflicts between meeting deadlines and quality)	Yes	*n* = 203 (73.6%)	15.175 *p* < 0.005	*n* = 162 (58.7%)		11.301 *p* < 0.005			*p* > 0.05
	No	*n* = 124 (57.7%)		*n* = 98 (45.6%)					
**Stress factors from the job role**									
The lack of recognition	Yes	*n* = 188 (74.6%)	16.498 *p* < 0.001	*n* = 152 (60.3%)		11.68 *p* < 0.005			*p* > 0.05
	No	*n* = 139 (58.2%)		*n* = 108 (45.2%)					
Tasks for which there is a lack of sufficient qualification	Yes	*n* = 169 (75.1%)	14.546 *p* < 0.005	*n* = 131 (58,2%)		8.670 *p* < 0.05	*n* = 152	(67.6%)	0.585 *p* > 0.5
	No	*n*= 158 (59.4%)		*n* = 129 (48.5%)			*n* = 179	(67.3%)	
**Risks from the work environment**									
Adverse effects of work environment factors	Yes	*n* = 151 (78.6%)	25.284 *p* < 0.001	*n* = 121 (63%)		17.333 *p* < 0.001	*n* = 112	(58.3%)	15.08 *p* < 0.005
	No	*n* = 176 (58.9%)		*n* = 139 (46.5%)			*n* = 219	(73.2%)	
**Risks from the social environment at work**									
Lack of support from colleagues and leaders	Yes	*n* = 111 (77.6%)	11.502 *p* < 0.005	*n* = 95 (66.4%)		17.564 *p* < 0.001			*p* > 0.05
	No	*n* = 216 (62.1%)		*n* = 165 (47.4%)					
The psychosocial work environment	Yes	*n* = 97 (86.6%)	26.372 *p* < 0.001	*n* = 78 (69.6%)		18.914 *p* < 0.001	*n* = 66	(58.9%)	11.344 *p* < 0.005
	No	*n* = 230 (60.7%)		*n* = 182 (48%)			*n* = 265	(69.9%)	
Social and communication skills	Yes	*n* = 116 (80%)	18.230 *p* < 0.001	*n* = 104 (71.7%)		30.303 *p* < 0.001	*n* = 84	(57.9%)	10.566 *p* < 0.01
	No	*n* = 211 (61%)		*n* = 156 (45.1%)			*n* = 247	(71.4%)	

## Data Availability

The original contributions presented in this study are included in the article. Further inquiries can be directed to the corresponding authors. The data are not publicly available due to privacy and ethical restrictions.

## Abbreviations

The following abbreviations are used in this manuscript:

MBI-HSS(MP)	Maslach Burnout Inventory Human Services Survey
EE	Emotional Exhaustion
DP	Depersonalization
PA	Personal Accomplishment
WHO	World Health Organization
ICD	International Classification of Diseases
RHI	Regional Health Inspectorates
